# The Knowledge, Attitudes, and Associated Factors Regarding First Aid Among the General Public in Saudi Arabia

**DOI:** 10.7759/cureus.41387

**Published:** 2023-07-05

**Authors:** Kadeja A Bashekah, Reem Alqahtani, Abdulrahman M Aljifri, Saif Y Ashram, Essam Alghamdi, Amjad M Khallaf, Ziad A Ibrahim, Ibraheem M Ghulman, Meshal Alsudais, Abdulaziz W Banaja

**Affiliations:** 1 Diabetes and Endocrinology, Ministry of Health, Jeddah, SAU; 2 Family Medicine, King Abdulaziz University Hospital, Jeddah, SAU; 3 Medicine, King Abdulaziz University Hospital, Jeddah, SAU; 4 Medicine, King Abdulaziz University, Jeddah, SAU

**Keywords:** public, saudi arabia, knowledge, first aid, attitude

## Abstract

Background: Accidents may occur at any time and in any location. Unintentional accidents may have life-threatening consequences. Anyone with basic first aid knowledge can assess the situation and intervene to provide proper care. This research aims to assess public knowledge and attitudes toward first aid and its related aspects in Saudi Arabia.

Methods: A cross-sectional online survey was conducted between April and May 2023 to investigate first aid knowledge, attitude, and associated factors among the general public of Saudi Arabia. This study's population consisted of Saudi Arabians aged at least 18 who are part of the general community. This research adapted and used a previously developed questionnaire to evaluate the general public's knowledge, attitudes, and other characteristics about first aid in Saudi Arabia. A binary logistic regression analysis was utilized to determine the variables that influence their knowledge and attitude.

Results: A total of 1135 participants were involved in this study. Almost one-third of the study participants (36.0%) reported that they had received training in first aid. The vast majority of the study participants (94.5%) reported that they had heard of first aid before. The media was the most commonly reported source of information on first aid (37.6%). Choking (63.2%), breathing difficulty (61.7%), and fainting (56.7%) were the most commonly reported indications (injuries or accidents) that need first aid. The mean knowledge score for the study participants was 4.4 (SD: 2.8) out of 8 (55.0%), which represents a moderate level of knowledge of first aid. The mean attitude score for the study participants was 22.5 (SD: 2.7) out of 28 (80.4%), which reflects a positive attitude toward first aid. Binary logistic regression analysis identified that women, those who have a higher education level, medical students, those with a high monthly income (7500 Saudi Arabian rials (SAR) and above), and those who work in the healthcare sector were more likely to be knowledgeable about first aid (p<0.05). Participants aged 31 to 40 years and university students were more likely to have a positive attitude toward first aid (p<0.05).

Conclusion: This research highlights the need to educate the public about first aid and emergency treatment. Even though one-third of participants have received first aid training, ongoing training is necessary. First aid information, especially on social media, is often unreliable. Choking, difficulty breathing, and syncope are typical first aid conditions, and awareness of dealing with choking is needed. Gender, socioeconomic status, and education influenced first aid knowledge and attitudes. Women, medical students, and healthcare workers knew more about first aid. Most participants supported first aid provision. This research strongly suggests improving awareness, providing inexpensive first-aid training, and targeting specific populations to improve first-aid knowledge and attitudes.

## Introduction

Accidents need quick and proper life-saving care before a patient receives substantial therapy [1]. This life-saving intervention known as 'first aid' consists of an examination and actions that may be administered promptly by an individual who is on site of the accident and with minimal or no medical equipment [2]. Thus, it is essential to possess a fundamental understanding of first aid. The ultimate objective of first aid is to prevent or reverse any potential injury before reaching the right medical facility [3]. Knowledge of first aid consists of procedures and strategies for quick response in health crises. It may be administered in all settings, including at home, school, business, and recreational locations. Knowledge of first aid also improves social responsibility and develops values [4]. According to a study, two-thirds of children have at least one unexpected injury every year. The majority of unintended injuries happened to children whose parents did not feel it was feasible to avoid them [5]. Unintended child injuries at home may originate from the impression that certain injuries are a normal component of child development [6]. Another study revealed that young children from low-income homes are more susceptible to accidental injuries [7]. Messages on injury avoidance should be simple, clear, and presented in both textual and visual formats. The importance of social networks in increasing knowledge and adherence to child safety guidelines [8]. Around 3% of families reported having at least one member who was wounded or died in the preceding year. Urban individuals are more likely to sustain an injury than their rural counterparts. One-third of all home injuries involve children less than nine years old [9]. Children, whose bodies are still growing and developing, are prone to unintentional injury.

Few studies on the public's knowledge and attitude toward first aid have been conducted. Prior research has focused on various demographics, notably teachers and university students [10-12]. A study in Spain examined the knowledge of first aid among primary and preschool teachers and the parents of children [13]. This study found that 57% of the participants had knowledge of first aid. Only four participants were able to define the basic life support sequence, and no one was able to successfully answer the cardiopulmonary resuscitation questions [13]. Another study in Saudi Arabia examined maternal knowledge and attitudes regarding first aid among their children [14]. In this study, the researcher found that two-thirds (65.5%) and 69.8% of the mothers who participated in the study had inaccurate information regarding the definition of first aid and its components, respectively [14]. Moreover, almost two-thirds (67.4%) were inaccurate regarding first aid for burns. Less than half (41.5% and 45%) knew the proper first aid procedures for treating nasal bleeding and fractured bones, respectively [14].

As the focus of this research is on the knowledge, attitude, and related variables of the general population about first aid, the findings would serve as vital information for various organizations. The results of this research may be used to promote life-saving practices among the general population of Saudi Arabia.

## Materials and methods

Study design, settings, and sampling procedure

A cross-sectional online survey was conducted in Saudi Arabia between April and May 2023 to investigate knowledge of first aid, attitudes toward it, and associated factors among the general public. This investigation's sample was selected using a process known as convenience sampling. This study involved patients who met our inclusion criteria and were willing to participate. On the first page of the questionnaire, participants were provided with an informed consent form and given the choice to continue participating in the study or withdraw from it. To ensure that applicants comprehended the relevance of their participation, the research's aims were communicated in full. The inclusion criteria were outlined in the invitation letter for the study.

Study population and recruitment

The inclusion criteria for the study's population consisted of Saudi Arabians at least 18 years old who are part of the general community. There were no limits on age or gender. The survey link was published on social media channels to encourage participation.

Study tool

This research adapted and used a previously developed questionnaire by Ganfure et al. to evaluate the general public's knowledge, attitudes, and other characteristics about first aid in Saudi Arabia [10]. The questionnaire (see Appendices) inquired about the respondent's age, gender, level of education, smoking status, the existence of comorbidities, and occupation. Twelve questions were used to assess the respondents' knowledge of first aid based on whether or not they had heard of or had training in first aid. They were also asked about the source of their knowledge. The knowledge score was determined based on the participant's responses to eight questions (in the form of yes/no questions) that tested their knowledge of basic first aid procedures. Participants received one point for each accurate response, with a maximum possible score of eight. The knowledge questions have been adapted from Pediatric First Aid for Caregivers and Teachers (PedFACTs) [15].

Participants attitudes about first aid were assessed using seven questions. Their responses were evaluated using a four-point Likert scale ranging from 1 (strongly disagree) to 4 (strongly agree) on a scale of 4. The maximum possible score will be 28. The higher the score, the more likely it is that the attitude toward first aid was positive. The development of questions on attitude followed previous literature [5,16,17]. In addition, the questionnaire was evaluated using the content validity index technique [18].

Piloting of the questionnaire tool

Doctors from the Saudi Ministry of Health and King Abdulaziz University's School of Medicine assessed and verified the questionnaire. Participants were asked about the clarity, comprehensibility, and face validity of the questions, and if any were difficult to understand. Additionally, participants were asked if they found any questions disrespectful or annoying. They said that the questionnaire was straightforward to understand and complete. Also, pilot research was done with a small sample of the study population to evaluate their grasp of the questionnaire prior to its widespread adoption.

Survey translation

To promote the involvement of the general population in Saudi Arabia, the questionnaire was translated into Arabic utilizing a forward-backward translation technique. The translation process ensured that the translated questionnaire maintains the same meaning and context as the original one.

Sample size and ethical approval

The minimum required sample size was 385 individuals using a 95% confidence interval (CI), a 0.5 standard deviation (SD), and a 5% margin of error. This study was approved by the Institutional Review Board of the Ministry of Health, Jeddah, Saudi Arabia (approval no. A01611). As participation in the study was voluntary, the research ethics committee approved the consent waiver.

Statistical analysis

This study's data were analyzed using SPSS Statistics version 27 (IBM Corp., Armonk, NY, USA). A histogram and normality metrics were used to examine the normality of the knowledge score. Based on the normality of the data, we presented the knowledge and attitude score as the mean (SD). In a binary logistic regression analysis, the mean knowledge and attitude scores of the patients were utilized as the dummy variable to determine the variables that influence their knowledge and attitude. To determine statistical significance, a two-sided p-value less than 0.05 was utilized.

## Results

Demographic characteristics of participants

A total of 1135 participants were involved in this study. More than half of them (64.4%) were women and married (68.2%). Almost one-third of the study participants (33.2%) were aged 30 years or younger. Almost half of them (49.3%) reported that they hold a bachelor's degree. The monthly income category was 7500 Saudi Arabian riyals (SAR) and above for 55.9% of the participants. Around 31% of the participants were unemployed. The vast majority of the participants (85.5%) were Saudi. A total of 16.4% of the participants reported that they are current smokers, and 38.7% reported that they have a history of chronic disease. Table 1 presents the demographic characteristics of the study participants.

**Table 1:** Demographic characteristics of participants SAR: Saudi Arabian rial

Variables	Frequency	Percentage
Gender		
Female	731	64.4%
Age		
18 to 23 years	139	12.2%
24 to 30 years	125	11.0%
31 to 40 years	184	16.2%
41 to 50 years	238	21.0%
51 to 60 years	275	24.2%
61 years and over	174	15.3%
Level of education		
Medical student	33	2.9%
Non-medical student	31	2.7%
High school or lower	244	21.5%
Diploma	134	11.8%
Bachelor	560	49.3%
Higher education	133	11.7%
Marital status		
Single	261	23.0%
Married	774	68.2%
Divorced	66	5.8%
Widowed	34	3.0%
Monthly income level		
Less than 2500 SAR	193	17.0%
2500-5000 SAR	173	15.2%
5000-7500 SAR	134	11.8%
7500 SAR and above	635	55.9%
Employment status		
Unemployed	349	30.7%
Retired	268	23.6%
Work in the healthcare sector	115	10.1%
Work outside the healthcare sector	285	25.1%
Student	118	10.4%
Nationality		
Saudi	970	85.5%
Are you a current smoker? (Yes)	186	16.4%
Do you have any chronic diseases? (Yes)	439	38.7%

Public knowledge of first aid

Table 2 presents participants' responses to items that examined public knowledge of first aid. Almost one-third of the study participants (36.0%) reported that they had training in first aid. The vast majority of the study participants (94.5%) reported that they had heard of first aid before. Media was the most commonly reported source of information on first aid (37.6%). Choking (63.2%), breathing difficulty (61.7%), and fainting (56.7%) were the most commonly reported indications (injuries or accidents) that need first aid.

**Table 2:** Responses to the questionnaire assessing public knowledge of first aid

Variables		Frequency	Percentage
Do you have training in first aid? (Yes)		409	36.0%
Have you ever heard about first aid? (Yes)		1073	94.5%
If yes to the previous question, from where did you hear about first aid? (You can choose more than one option) (n= 1073)			
Media		403	37.6%
Health professionals		187	17.4%
Health institution		173	16.1%
Books		161	15.0%
Family		149	13.9%
If yes to the previous question, what is first aid? (n= 1073)			
The immediate care given to a person who sustains an injury or accident, and before the victim arrives at a health institution.		755	70.4%
The care given only in health institutions		26	2.4%
The care given only by health professionals		20	1.9%
Don’t know		272	25.3%
What type of injuries/accidents need first aid? (You can choose more than one option)			
Choking		717	63.2%
Breathing difficulty		700	61.7%
Fainting		643	56.7%
Bleeding		614	54.1%
Burning		602	53.0%
Nose bleeding		591	52.1%
Epilepsy		487	42.9%
Fracture		453	39.9%
Swallowed poison		438	38.6%
Human/animal bite		400	35.2%
Neck and back injury		329	29.0%
Answer the following	True	False	Don’t know
“One measure to stop bleeding is pressing firmly with a clean bandage on the part that is bleeding"	81.5%	5.6%	12.9%
“Giving nothing by mouth is one of the first aid measures for a fainting child”	70.4%	8.7%	20.8%
“One of the first aid measures for an epileptic child is keeping the airway clear by placing the child on his/her side”	61.3%	4.4%	34.4%
“Standing behind the child encircling the child’s chest with hands and squeezing is the first aid measure for a choking child”	85.7%	3.6%	10.8%
“For a child with neck and back injury, avoiding head and neck movement and keeping the body straight is one measure of first aid.”	83.9%	1.0%	15.1%
“When a child is been bitten by a friend, cleansing the wound with soap and water for 5 minutes is one measure of first aid for a human bite.”	31.9%	21.3%	46.8%
“One of the first aid measures for nose bleed/epistaxis is placing the student in a comfortable sitting position while slightly leaning forward and applying uninterrupted pressure by pressing the nostrils together.”	61.1%	12.9%	26.0%
“Encouraging the child to sit quietly, breath slowly and deeply in through the nose and out through the mouth is first aid measure for a child with breathing difficulty"	70.5%	2.7%	26.8%

The proportion of right answers for knowledge items ranged between 31.9% and 85.7%. Around 85.7% of the participants signaled their awareness that "standing behind the child, encircling the child’s chest with hands, and squeezing is the first aid measure for a choking child." Only 31.9% knew that "when a child is bitten by a friend, cleansing the wound with soap and water for five minutes is one measure of first aid for a human bite."

Public attitude toward first aid

**Figure 1:**
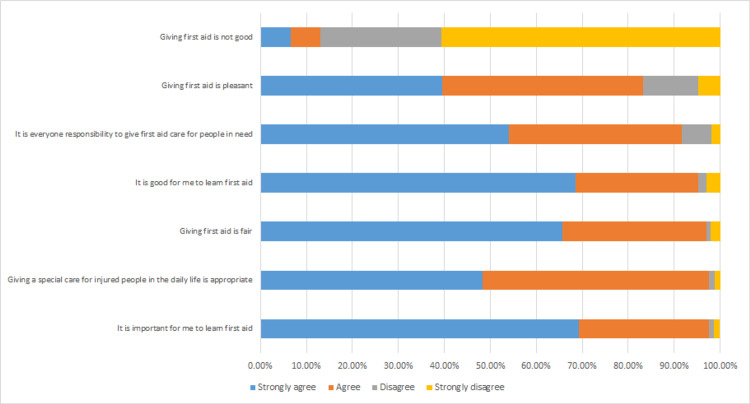
Public's attitude toward first aid

Figure 1 illustrates the public's attitude toward first aid. Agreement levels (participants who answered agree or strongly agree) toward first aid provision ranged between 13.1% and 97.6%. The most commonly agreed upon items were: it is important to learn first aid (97.60%), giving special care to injured people in daily life is appropriate (97.50%), and giving first aid is fair (97.10%).

Predictors of knowledge and positive attitude in participants

The mean knowledge score for the study participants was 4.4 (SD: 2.8) out of 8 (55.0%), which represents a moderate level of knowledge of first aid. The mean attitude score for the study participants was 22.5 (SD: 2.7) out of 28 (80.4%), which reflects a positive attitude toward first aid. Binary logistic regression analysis identified that women, those who have higher education levels, medical students, those with high monthly income (7500 SAR and above), and those who work in the healthcare sector were more likely to be knowledgeable of first aid (p<0.05). Participants aged 31 to 40 years and university students were more likely to have a positive attitude toward first aid (p<0.05) as seen in Table 3.

**Table 3:** Binary logistic regression analysis *p<0.05; **p<0.01; ***p<0.001 OR: Odds ratio, CI: Confidence interval, SAR: Saudi Arabian rial

Variables	OR of having better knowledge (95% CI)	OR of having a more positive attitude (95% CI)
Gender		
Female (reference category)	1.00	
Male	0.74 (0.58-0.95)*	0.79 (0.61-1.01)
Age		
18 to 23 years (reference category)	1.00	
24 to 30 years	1.03 (0.71-1.50)	1.00 (0.69-1.46)
31 to 40 years	1.33 (0.96-1.84)	1.43 (0.104-1.96)*
41 to 50 years	1.01 (0.75-1.34)	1.19 (0.89-1.59)
51-60 years	1.01 (0.77-1.33)	0.72 (0.54-0.95)*
61 years and above	0.65 (0.47-0.90)**	0.64 (0.46-0.90)*
Level of education		
High school or lower (reference category)	1.00	
Bachelor	0.99 (0.78-1.25)	0.96 (0.76-1.21)
Higher education	2.20 (1.48-3.28)***	1.31 (0.91-1.88)
Diploma	0.86 (0.60-1.24)	1.16 (0.81-1.67)
Medical student	2.97 (1.28-6.91)*	1.33 (0.67-2.67)
Non-medical student	1.24 (0.60-2.58)	1.33 (0.65-2.71)
Marital status		
Single (reference category)	1.00	
Married	0.82 (0.64-1.05)	0.65 (0.51-0.84)***
Divorced	1.30 (0.78-2.16)	1.53 (0.93-2.52)
Widowed	0.54 (0.27-1.07)	0.66 (0.32-1.38)
Monthly income level		
Less than 2500 SAR (Reference category)	1.00	
2500-5000 SAR	0.84 (0.61-1.17)	0.95 (0.68-1.32)
5000-7500 SAR	0.99 (0.69-1.42)	1.01 (0.70-1.46)
7500 SAR and above	1.32 (1.04-1.67)*	1.04 (0.82-1.32)
Employment status		
Retired (Reference category)	1.00	
Unemployed	0.76 (0.59-0.98)*	0.86 (0.66-1.11)
Work in the healthcare sector	2.94 (1.87-4.62)***	1.28 (0.87-1.88)
Student	1.47 (0.99-2.18)	1.58 (1.08-2.31)*
Work outside the healthcare sector	0.96 (0.73-1.26)	1.09 (0.83-1.43)
Nationality		
Saudi (reference category)	1.00	
Non-Saudi	0.77 (0.55-1.08)	1.18 (0.85-1.66)
Are you a current smoker? (Yes)	0.99 (0.72-1.35)	1.22 (0.89-1.68)
Do you have any chronic diseases? (Yes)	0.84 (0.66-1.07)	0.73 (0.57-0.93)*

## Discussion

The prevalence of accidents and their life-threatening consequences necessitate urgent, immediate, and appropriate lifesaving care [1]. This can be accomplished via an assessment and intervention by any nearby individual with basic first-aid knowledge [2]. Consequently, the purpose of this study was to evaluate the general public's knowledge, attitudes, and associated factors regarding first aid in Saudi Arabia.

Nearly one-third of the study participants (36.0%) reported having first aid training, which is greater than the percentage required to assure the presence of assistance, where at least 30% of the population should have first aid knowledge [19]. Moreover, 94.5% of the study participants reported having heard of first aid, which is a positive indicator of the importance of first aid training and knowledge among the general public [10,20]. Indeed, the public plays a significant role in saving lives during emergencies, but this can only be accomplished if the public is well-trained and has the right knowledge to intervene at the scene of an emergency [21]. However, this training and knowledge must be kept up-to-date, as skills can deteriorate over time without retraining [22]. Typically, first aid training is valid for three years, and annual refresher training is recommended [23]. 

The most commonly reported source of information on first aid was the media, which accounted for 37.6% of the responses. While the use of social media as a source of information has increased significantly in recent years, its credibility is questionable since information on it can be controlled by its users [24]. However, the presence of approved information and instructions on media platforms will increase the public's knowledge, which will aid in the reduction of first aid-related deaths. 

Choking, difficulty breathing, and fainting were the most frequently reported injuries or accidents requiring first aid (63.2%, 61.7%, and 56.5%, respectively). Choking is one of the leading causes of unintentional injury-related death; however, not everyone is aware of the first aid procedures for such cases, and therefore, there is a need to raise awareness of choking first aid procedures [25]. Meanwhile, breathing difficulty is one of the most common reasons for an ambulance response, and there are multiple protocols and procedures available to treat patients with breathing difficulties [26]. However, fainting is well understood by the general public, and it is common in humans with multiple causes, but the issue is the lack of concern regarding fainting, where people may believe that the fainter is seeking attention or because they believe that those who faint in their presence recover instantly [27].

This study revealed that 85.7% are familiar with "standing behind the child, encircling the child's chest with your hands, and squeezing is the first aid measure for a choking child" or the Heimlich maneuver that aims to open the airway of the choking person [28]. And for mothers, this study's results are not consistent with any other study that demonstrates a lack of knowledge of first aid. Only 13.9% were aware that "if a child is bitten by a friend, cleaning the wound with soap and water for 5 minutes is one measure of first aid for a human bite." This low level of awareness may be due to the underestimation of a human bite and its consequences [29]. Infections occur in 10% to 15% of all human bites and are the third leading cause of all bites seen in hospital emergency rooms [30], highlighting the need to increase awareness of human bites and their consequences.

In fact, gender, socioeconomic factors, and educational background influence the percentage of knowledge and attitude toward first aid. Among the study participants, the mean knowledge of first aid was 55%, which indicates a moderate level of first aid knowledge. In addition, the study found that women, those with a higher level of education, those with a high monthly income, medical students, and those who work in the healthcare industry were more likely to have first aid knowledge. In the meantime, women and health care professionals are more likely to be subjected to first aid courses and training [31], resulting in this high first aid knowledge score. However, an individual's income and the availability of a local first aid course play a significant role in learning first aid [32,33], with low-income families citing course costs as a primary barrier to learning first aid [33]. Nonetheless, in this study, the majority of participants (80.4%), those aged 31 to 40 years, and university students have a positive attitude toward first aid. These findings are similar to those of other studies that demonstrate an increase in positive attitudes toward first aid by knowing the importance and usefulness of learning and providing first aid [10,17]. Furthermore, exposure of university students and individuals aged 31 to 40 years to first aid training increased the likelihood that they had a positive attitude towards first aid and its application [34,35].

There could be a number of factors that contribute to the perception that women are more interested in first aid. Women are more likely than men to be interested in and knowledgeable about first aid due to factors including socialization, professional roles, and community involvement. To encourage men to become more knowledgeable and interested in first aid, it is essential to create an inclusive and accessible environment that promotes the benefits and relevance of first aid skills for everyone. Launching awareness campaigns that specifically target men is an important and recommended strategy.

This study has limitations. A significant proportion of the targeted population may not have access to social media websites, so the use of an online survey to recruit study participants is not without criticism. However, according to the most recent available statistics from 2023, approximately 79.3 percent of the Saudi Arabian population uses social media. Our ability to observe causal relationships between study variables was limited by the cross-sectional study design. Our study's findings may be less generalizable due to the technique of convenient sampling that was utilized.

## Conclusions

This study emphasizes the critical need for appropriate emergency care in emergencies and the significance of first aid knowledge and intervention by the general public. Despite the fact that one-third of the participants had received first aid training, continuous training, and updates are required. The media, especially social media, have become a common source of information on first aid, but the veracity of this information is questionable. Choking, breathing difficulty, and syncope were identified as common situations requiring first aid, with a need to raise awareness regarding choking procedures in particular. Gender, socioeconomic factors, and educational background affected the level of knowledge and attitudes regarding first aid. Women, those with a higher level of education and income, medical students, and healthcare professionals exhibited greater levels of knowledge. on first aid. The majority of participants exhibited favorable attitudes regarding first aid. This study emphasizes the significance of raising awareness, providing accessible and affordable first aid courses, and targeting specific groups to improve knowledge of and attitudes toward first aid.

## Appendices

Questionnaire

Participant Information Sheet

Title of the research project: “First aid knowledge, attitudes, and associated factors among the general public in Saudi Arabia”.

First of all,  we would like to thank you in advance for your cooperation and consent to participate in this study. Please read the general information on this study. If you have any questions regarding the study please ask freely.

The results of the study will be used as baseline information to design appropriate intervention strategies to increase public knowledge and attitude toward first aid. The questionnaire contains both closed-ended questions and will be provided in a self-administered online form. You are therefore kindly requested to provide genuine answers to the questions. The information you provide is confidential and is used only for the purpose of this study. If you have any questions, don‘t hesitate to ask the data collector. Your cooperation and participation until the completion of the questionnaire is very necessary for the successful completion of the study.

We, therefore, ask for your genuine willingness. However, you have the right to refuse if you are not willing to participate by making a tick mark on ‘No’ in the box below.

Are you volunteering?         Yes                                            No

Thank you in advance for your cooperation

Name of data collector: ____________________, date: ____________, signature: __________

Questionnaire code: _________________

English Version of the Questionnaire

**Table 4:** Study questionnaire tool

No.	Sociodemographic details	Response	Remark
1	Sex	Male/Female	
2	Age		
3	Level of education	Certificate/Diploma Degree/Masters/Other (specify) ____	
4	Marital status	Married/Single/Divorced/Widowed	
5	Do you have training in first aid?	Yes/No	

1. Have you ever heard about first aid?
a. Yes
b. No
2. If you answered Yes to Q1, from where did you hear of first aid? You can choose more than one option.
a. Family
b. Books
c. Media
d. Health professionals
e. Health institution
f. Others, specify___________________
3. If you answered Yes to Q1, what is first aid?
a. The immediate care given to a person who sustains any injury or accident, and before the injured person arrives at a health institution.
b. The care given only in health institutions
c. The care given only by health professionals
d. Others, specify ________________________________________
4. What type of injuries/accidents need first aid? You can choose more than one option
a. Bleeding
b. Fracture
c. Epilepsy
d. Human/animal bite
e. Burning
f. Nose bleed
g. Choking
h. Neck and back injury
i. Fainting
j. Swallowing of poison
k. Breathing difficulty
l. Others, specify ________________________________________
5. One measure to stop bleeding is by pressing firmly with a clean bandage on the bleeding part
a. True
b. False
c. Don’t know
6. Giving nothing by mouth is one of the first aid measures for a fainting child
a. True
b. False
c. Don’t know
7. One of the first aid measures for an epileptic child is keeping the airway clear by placing the child on his/her side
a. True
b. False
c. Don’t know
8. Standing behind the child encircling the child’s chest with hands and squeezing is the first aid measure for a choking child
a. True
b. False
c. Don’t know
9. For a child with neck and back injury, avoiding head and neck movement and keeping the body straight is one measure of first aid.
a. True
b. False
c. Don’t know
10. When a child has been bitten by a friend, cleansing the wound with soap and water for 5 minutes is one measure of first aid for a human bite.
a. True
b. False
c. Don’t know
11. One of the first aid measures for nose bleed/epistaxis is placing the student in a comfortable sitting position with a slight forward lean and applying uninterrupted pressure by pressing the nostrils together.
a. True
b. False
c. Don't know
12. Encouraging the child to sit quietly, and breathe slowly and deeply in through the nose and out through the mouth is a first aid measure for a child with breathing difficulty.
a. True
b. False
c. Don’t know
Respond to the following statements with the given choices

1. Giving first aid is fair	Strongly agree/Agree/Disagree/Strongly disagree
2. Giving first aid is pleasant	Strongly agree/Agree/Disagree/Strongly disagree
3. Giving first aid is not good	Strongly agree/Agree/Disagree/Strongly disagree
4. It is good for me to learn first aid	Strongly agree/Agree/Disagree/Strongly disagree
5. It is important for me to learn first aid	Strongly agree/Agree/Disagree/Strongly disagree
6. It is everyone’s responsibility to give first aid care for people in need	Strongly agree/Agree/Disagree/Strongly disagree
7. Giving special care for injured people in everyday life is appropriate	Strongly agree/Agree/Disagree/Strongly disagree
